# Causal Intervention and Counterfactual Reasoning for Multimodal Pedestrian Trajectory Prediction

**DOI:** 10.3390/jimaging11110379

**Published:** 2025-10-28

**Authors:** Xinyu Han, Huosheng Xu

**Affiliations:** 1 School of Computer Science and Technology, Harbin Engineering University, Harbin 150001, China; xuhuosheng@hrbeu.edu.cn; 2 Wuhan Digital Engineering Institute, Wuhan 430205, China

**Keywords:** trajectory prediction, causal intervention, multimodal prediction, feature fusion

## Abstract

Pedestrian trajectory prediction is crucial for autonomous systems navigating human-populated environments. However, existing methods face fundamental challenges including spurious correlations induced by confounding social environments, passive uncertainty modeling that limits prediction diversity, and bias coupling during feature interaction that contaminates trajectory representations. To address these issues, we propose a novel Causal Intervention and Counterfactual Reasoning (CICR) framework that shifts trajectory prediction from associative learning to a causal inference paradigm. Our approach features a hierarchical architecture having three core components: a Multisource Encoder that extracts comprehensive spatio-temporal and social context features; a Causal Intervention Fusion Module that eliminates confounding bias through the front-door criterion and cross-attention mechanisms; and a Counterfactual Reasoning Decoder that proactively generates diverse future trajectories by simulating hypothetical scenarios. Extensive experiments on the ETH/UCY, SDD, and AVD datasets demonstrate superior performance, achieving an average ADE/FDE of 0.17/0.24 on ETH/UCY and 7.13/10.29 on SDD, with particular advantages in long-term prediction and cross-domain generalization.

## 1. Introduction

Pedestrian trajectory prediction is a cornerstone technology for enabling safe and reliable autonomous systems, such as self-driving cars and social robots, which must navigate seamlessly within human-populated environments [1]. The core challenge lies in accurately forecasting a pedestrian’s future path based on their observed past motion and the surrounding social and physical context. While humans perform this task with intuitive ease, computational models must grapple with the inherent uncertainties of human decision-making [2], which are influenced by a complex interplay of personal goals, social norms, and environmental constraints [3,4].

Driven by the success of deep learning, significant progress has been made in this field. Early approaches [5] focused on modeling the social interactions between pedestrians using techniques like Social LSTM and graph neural networks. Subsequent work incorporated physical scene information through semantic maps or occupancy grids [6], and generative models like Generative Adversarial Networks [3] and Variational Autoencoders were introduced to produce multimodal predictions [7,8], acknowledging that a pedestrian’s future is not deterministic but a distribution of possibilities [9].

**Figure 1:**
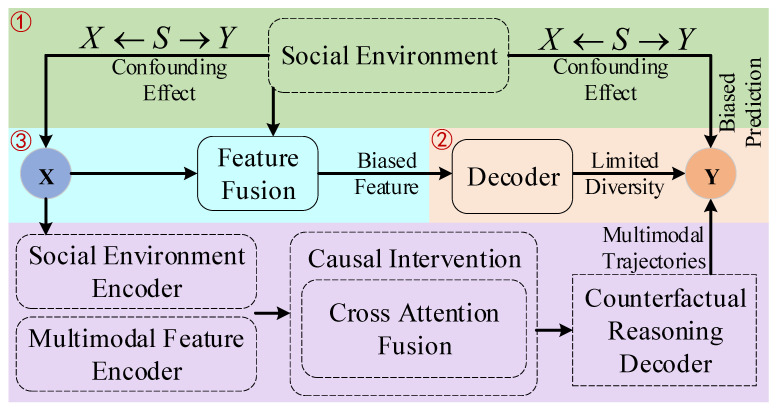
Existing challenges and proposed method.

Some existing approaches [10,11,12,13] regard social environmental influences as valuable contextual cues and attempt to capture these environmental correlations, but such methods face fundamental limitations from a causal inference standpoint. The social environment frequently functions as a confounding factor that concurrently affects both the pedestrian’s historical trajectory (X) and future trajectory (Y), establishing a back-door path X←S→Y as depicted in Figure 1 (green block). Consequently, even in the absence of direct causal relationships between specific historical trajectories *X* and future trajectories *Y*, the social environment *S* can introduce spurious correlation between them. Consider crowded intersection scenarios where pedestrians exhibit similar parallel movement patterns—this alignment often results from environmental constraints like narrow passages rather than intentional social interactions. Models dependent purely on likelihood estimation frequently misinterpret these environmentally induced coincidental patterns as social interactions between pedestrians [14], causing prediction inaccuracies when environmental conditions change. Current research [15,16] still lacks comprehensive understanding and effective mechanisms to mitigate this confounding effect.

Furthermore, as shown in Figure 1 (the orange block), although contemporary generative models [11,13] demonstrate multimodal prediction capabilities, their diversity primarily relies on stochastic sampling from latent distributions or extracting common patterns from training datasets [17]. This modeling paradigm remains essentially passive and retrospective in nature. Such models are constrained to extrapolate within patterns derived from historical observational data, lacking the capacity for active reasoning about potential future occurrences. This limitation becomes particularly evident when handling novel or counterfactual scenarios insufficiently represented in training data. For example, when a previously straight-walking pedestrian encounters an unexpected obstacle, the existing models [7,8,18] struggle to proactively reason about such plausible yet previously unobserved avoidance behaviors. Therefore, a more proactive uncertainty modeling mechanism that goes beyond mere historical data extrapolation is needed.

Additionally, in traditional feature fusion methods [19,20,21,22], such as simple concatenation or attention mechanisms, historical trajectory features and social environment features are typically interacted with without distinction. This leads to biased information learned from the social environment, carrying the aforementioned spurious associations, directly contaminating the representation of the historical trajectory, as shown in Figure 1 (the blue block). The model ultimately learns a feature representation entangled with both genuine causal signals and spurious correlative signals, undermining its generalizability and interpretability [23]. This problematic fusion process, leading to a biased feature representation, is highlighted as a key issue that our causal intervention module is designed to rectify.

To address the aforementioned challenges, this paper proposes a novel Causal Intervention and Counterfactual Reasoning (CICR) framework for multimodal pedestrian trajectory prediction. As illustrated in Figure 1 (the purple block), our core idea is to shift the prediction task from the traditional associative learning paradigm to a causal inference paradigm.

The proposed framework employs a hierarchical architecture comprising three specialized modules. The Multisource Encoder tackles the bias coupling issue by separately extracting trajectory features through a GRU and social context features through interaction modeling, preventing direct contamination between modalities. The Causal Intervention-based Feature Fusion Module specifically addresses spurious correlations by implementing front-door criterion through cross-attention mechanisms, generating deconfounded trajectory representations that capture genuine causal relationships rather than environmental biases. Finally, the Counterfactual Reasoning Decoder overcomes passive uncertainty modeling by simulating hypothetical scenarios through a policy network and multi-head attention, enabling proactive reasoning about diverse future possibilities. This comprehensive framework establishes a new paradigm for trajectory prediction that not only systematically resolves the fundamental limitations of existing approaches but also achieves superior predictive performance across diverse scenarios.

Our contributions can be summarized as follows:

- We propose a Multisource Encoder Module. This module separately extracts robust multi-scale spatio-temporal features from the pedestrian’s own trajectory and comprehensive interaction and semantic features from the social environment, providing a solid foundation for subsequent causal analysis and fusion.
- We present a Causal Intervention Fusion Module. To eliminate spurious correlations, this module formally models the social environment as a confounder using a Structural Causal Model and implements the front-door criterion via a Cross-Attention Fusion mechanism. Its role is to perform causal intervention, yielding a deconfounded trajectory representation that captures the true causal relationship between historical and future paths.
- We introduce a Counterfactual Reasoning Decoder. This decoder moves beyond passive extrapolation by proactively simulating hypothetical future scenarios. It uses a multi-head attention mechanism to enable the target pedestrian’s motion intent to interact with sampled future path nodes, thereby generating diverse and plausible trajectory predictions that account for future uncertainty.
- Extensive experiments and comprehensive ablation studies are conducted on several human trajectory prediction benchmarks, including ETH [24], UCY [5], the Stanford Drone Dataset (SDD) [25], and the ActEV/VIRAT Dataset (AVD) [26], to evaluate the effectiveness of our proposed method and validate the contributions of its key components.

## 2. Related Works

### 2.1. Trajectory Prediction

Based on the observed movement path, the human trajectory prediction system is constructed to estimate future positions. Most current approaches regard trajectory prediction as a sequential forecasting task. For instance, methods such as social force models [27], Kalman filtering [28], and Markov processes [29] are employed to capture the motion characteristics of pedestrians. With the advancement and success of deep learning, deep learning-based methods have been extensively applied in the field of motion prediction [18,30,31]. For example, Social-LSTM [1] was developed with a Social aggregation layer, which is used to aggregate information related to interactions between neighboring pedestrians. Zheng et al. [32] focused on the interaction features among pedestrians. Furthermore, graph-based approaches have also emerged as a research focus in this domain [33]. Wang et al. [34] emphasized learning robust features in complex scenarios to improve prediction accuracy. Furthermore, anti-geometric distortion mechanism [35] can provide inspiration for enhancing the robustness of trajectory prediction under environmental disturbances or scene transformations. Recently, the proven effectiveness of the multi-head self-attention mechanism has driven its wide application across various fields [36]. Some studies have applied attention-based models to explore the trajectory prediction of crowds [37].

Although these methods have achieved remarkable success, they fundamentally rely on learning strong correlations from the observed data. When the training data contains false statistical correlations caused by the environment, the model will internalize them, resulting in poor performance when encountering new environments or rare scenarios during testing. This limitation has given rise to a new trend of re-examining trajectory prediction problems from a causal perspective.

### 2.2. Causal Inference

Causal inference [38] estimates causal effect under covariate shift [39], causal intervention cuts spurious cause–effect correlations to capture true causality, and related methods help deep networks learn true causalities [40] to reduce prediction error, using causal intervention to remove such correlations. As a key field across statistics, medicine, CS, and economics, causal reasoning has entered autonomous driving. Li [41] applies it to end-to-end driving model decision-making, but existing works only capture scene–trajectory correlations, and few study trajectory prediction via causality. Chen [42] uses counterfactual analysis to reduce training-deployment discrepancy for biased social environments. Liu [43] focuses on HTP robustness to noisy input. Huang [44] uses masking for counterfactual reasoning.

However, most of the existing methods mainly focus on “bias correction” within the scope of historical observations, and they have not fully addressed the issue of active reasoning for future uncertainties. This leaves room for innovation to introduce counterfactual reasoning into trajectory prediction, in order to enable the model to generate more reasonable future hypotheses.

### 2.3. Multimodal Prediction

To address the inherent unpredictability of human motion, a variety of stochastic prediction approaches has been developed to capture the randomness of future trajectories. Among them, a number of methods employ RNN or LSTM architectures to estimate trajectory probabilities by learning grid-based location encoders [3,20,30,34,45]. The introduction of Social GAN [3] marked a notable step by explicitly emphasizing multimodal trajectory prediction, establishing its importance in the field.

Alternative strategies have also been explored. Several methods utilize attention mechanisms to capture spatio-temporal features for stochastic prediction [18,36]. CGTP [6], for example, enhances collision priors to construct a multi-graph social encoder and employs pixel attention along with target response maps to optimize scene encoding. Similarly, Chen et al. [8] propose DSTIGCN, which models spatio-temporal interactions via deformable graph convolutional networks and uses Latin hypercube sampling to alleviate long-tail effects in multimodal prediction.

Another line of work adopts the CVAE framework to model multimodality [3,7,46,47]. In particular, Kim et al. [7] present HighGraph, which builds relational graphs using a collision-sensitive kernel and applies higher-order graph convolutions to aggregate social features across distances, thereby better capturing indirect higher-order social interactions.

The latest research frontiers are beginning to explore how to integrate counterfactual reasoning into the multimodal generation process. Some works [48,49] attempt to introduce counterfactual thinking into the trajectory prediction of autonomous driving, by imagining “if the agent takes different actions” to assess risks and generate safer plans. This indicates that multimodal prediction is evolving from “data-driven probability sampling” to “knowledge-guided active reasoning”, and our work is precisely exploring the frontier in this direction.

## 3. Methodology

### 3.1. Description of Symbols

For reference, a list of symbols used in this work is given in Table 1.

### 3.2. Architecture of CICR

**Figure 2:**
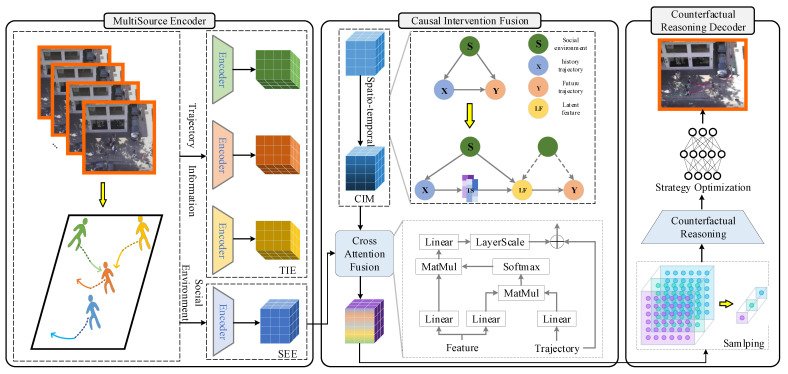
Architecture of the CICR model.

This work presents a novel pedestrian trajectory prediction framework that effectively addresses confounding bias from social environments and overcomes limitations in modeling future uncertainties through the integrated application of causal reasoning and counterfactual reasoning. As illustrated in Figure 2, our framework employs a hierarchical architecture consisting of three core components: a Multisource Encoder, a Causal Intervention Fusion Module, and a Counterfactual Reasoning Decoder.

The framework begins with the Multisource Encoder, which extracts multi-level features from raw input data through parallel processing branches. The trajectory encoding branch captures pedestrians’ intrinsic motion patterns via sequence modeling, while the environment encoding branch integrates social interactions and scene semantics, providing comprehensive feature representations for subsequent analysis.

Following this, the Causal Intervention Fusion Module eliminates spurious correlations induced by social environments through its two components: the CIM and CAF. By constructing causal graphs and employing attention mechanisms, this module performs feature-level causal interventions to obtain deconfounded trajectory representations, ensuring the learning of genuine causal relationships.

Subsequently, the Counterfactual Reasoning Decoder generates diverse trajectory predictions by simulating multiple plausible future scenarios. Through sampling potential future paths and enabling cross-modal interaction between historical features and hypothetical situations, this module produces multimodal predictions that maintain physical realism while enhancing prediction diversity.

The complete framework operates through an end-to-end training paradigm, establishing a coherent pipeline from feature extraction and causal de-biasing to multimodal prediction. This integrated approach significantly improves result diversity and interpretability while maintaining high prediction accuracy.

### 3.3. Problem Formulation

Given a sequence of the historical trajectory information of *n* pedestrians, including the historical locations of the $i^{th}$ pedestrian $O_{obs}^{i}=\left\{O_{1}^{i},\ldots ,O_{obs}^{i}\right\}=\left\{\left(x_{1}^{i},y_{1}^{i}\right),\ldots ,\left(x_{obs}^{i},y_{obs}^{i}\right)\right\}$ from time step 1 to obs, where $\left[1,\;obs\right]$ represents the historical observation of trajectory length and $O_{1}^{i}=\left(x_{1}^{i},y_{1}^{i}\right)$ denotes the first observed historical location of the $i^{th}$ pedestrian, the corresponding ground truth is denoted as $O_{pre}^{i}=\left\{O_{obs+1}^{i},\ldots ,O_{pre}^{i}\right\}=\left\{\left(x_{obs+1}^{i},y_{obs+1}^{i}\right),\ldots ,\left(x_{pre}^{i},y_{pre}^{i}\right)\right\}$. The vector coordinate of the $i^{th}$ pedestrian is represented as $v_{obs}^{i,mn}=\left(L_{obs}^{i,mn},\Theta_{obs}^{i,mn}\right)$ constructed by $O_{m}^{i}$ and $O_{n}^{i}$, where L denotes the polar radius value and Θ denotes the polar angle value, m,n∈[1,obs],m<n. The predictions of the $j^{th}$ location of the $i^{th}$ pedestrian are denoted as $v_{pre}^{i,\left|m-n\right|j}=\left(L_{pre}^{i,\left|m-n\right|j},\Theta_{pre}^{i,\left|m-n\right|j}\right)$ based on $v_{obs}^{i,mn}$. The prediction of the $i^{th}$ pedestrian is denoted as ${\bar{O}}_{pre}^{i}=\left\{O_{obs+1}^{i},\ldots ,O_{pre}^{i}\right\}=\left\{\left(x_{obs+1}^{i},y_{obs+1}^{i}\right),\ldots ,\left(x_{pre,}^{i}y_{pre}^{i}\right)\right\}$, where $\left[obs+1,\;pre\right]$ denotes the prediction length. A series of methods has been developed to predict future trajectories using relative position or velocity information [4]. The social interaction information of the $i^{th}$ pedestrian can be defined as a function of the trajectories of the surrounding pedestrians $\left(e_{i}=e\left({\tilde{O}}_{obs}^{i}\right)\right)$, where e(·) is a social information aggregation function (such as the social pooling in Social-LSTM), and ${\tilde{O}}_{obs}^{i}={\left[O_{obs}^{j}\right]}_{j=0,j\neq i}^{n}$. The formal definition of pedestrian trajectory prediction is as follows:

(1) $${\bar{O}}_{pre}^{i}=\Gamma \left(O_{obs}^{i},e_{i}\right)$$

where ${\bar{O}}_{pre}^{i}$ denotes the future trajectory predictied by the model Γ(·).

### 3.4. Multisource Encoder Module

The multisource encoding module primarily consists of two components: TIE and SEE. The pedestrian trajectory prediction task presents significant challenges, primarily stemming from strong temporal dependencies and the underutilization of historical features. Therefore, extracting more discriminative features from limited data is crucial for improving prediction accuracy. To address these issues, we propose a multi-scale encoding mechanism. This encoder first processes the features of the original trajectory data. To comprehensively describe pedestrian dynamic behavior, we extract multimodal features based on the input trajectory sequence, including spatio-temporal features and keypoint features of pedestrian motion. Specifically, the spatio-temporal features aim to capture the potential periodic patterns in pedestrian movement trajectories. As indicated by Autoformer [37], time series forecasting typically involves periodic patterns, and pedestrian trajectory prediction is no exception. Empirical observations show that pedestrian trajectories often exhibit slight arcs or approximate straight-line movements, reflecting a certain degree of regularity. Consequently, we construct the spatio-temporal feature representation by recording the sequential positional information for each frame from t=1 to $t=t_{\mathrm{pre}}$. Based on the annotated pedestrian positions in the dataset, we compute the corresponding temporal features, providing a foundation for subsequent feature fusion and causal inference. Furthermore, keypoint features are extracted from the videos using the pre-trained HRNet model [50] to obtain pedestrian keypoint information, which is then fused with the spatio-temporal features.

To achieve efficient encoding while maintaining prediction performance, we select a GRU as the core module of the encoder. The GRU exhibits excellent capability in modeling temporal dependencies and offers high computational efficiency, making it suitable for resource-constrained practical scenarios. Given that pedestrian motion inherently contains significant uncertainty, and such stochasticity is naturally present in the raw trajectory data, the model should directly learn the intrinsic dynamic variations within the data, rather than artificially suppressing random information through smoothing operations. To further capture short-term dependencies in both the temporal and feature dimensions, we employ average pooling to aggregate historical trajectory information within a sliding time window, thereby extracting more robust local dynamic features. To mitigate the potential loss of critical details caused by pooling operations, we introduce a linear transformation to remap the original input, achieving multi-scale information fusion. Subsequently, the feature dimensions are adjusted to generate a sequence representation that meets the input requirements of the GRU. Finally, the GRU encodes this feature sequence to extract long-range dependencies, completing the multi-level, sequential trajectory feature modeling.

(2) $$\tau =GRU(Linear\left(Avgpooling\left(O_{obs}^{i}\right)\right))$$

Traditional methods often rely on historical behavior and social interaction information for trajectory prediction. The social environment is a key confounding variable that affects prediction performance, primarily by inducing the model to learn spurious correlations between historical and future trajectories. Specifically, the social environment (denoted as variable *S*) simultaneously influences both the historical trajectory *X* and the future trajectory *Y* of a pedestrian, forming a back-door path X←S→Y. This means that even if no direct causal relationship exists between certain historical trajectories *X* and future trajectories *Y*, *S* can still introduce spurious statistical associations by influencing both. For example, in crowded scenarios, parallel movement between pedestrians might not stem from group behavior but rather from movement patterns constrained by the environment. If the model relies solely on likelihood estimation, it may incorrectly associate crowded environments with group behavior, thereby reducing prediction accuracy. To address this, our designed social environment encoder not only encodes the information of surrounding pedestrians but also integrates the semantic information of the scene to more comprehensively characterize the influence of the social environment.

### 3.5. Causal Intervention Fusion Module

The social environment exerts a significant confounding effect on future trajectory prediction. If not adequately addressed, this effect can mislead the model into learning spurious correlations between historical and future trajectories that do not genuinely exist, thereby severely compromising the reliability and accuracy of prediction performance. Consequently, prior to fusing features from different modalities, it is imperative to design effective mechanisms from a causal perspective to identify and eliminate these spurious statistical associations within the model.

#### 3.5.1. Structural Causal Model

To systematically analyze and formalize the aforementioned issue, we utilize a SCM, aiming to clearly characterize the intrinsic causal mechanisms between variables. This SCM comprises three core variables (nodes): *X* (denoting the historical trajectory), *Y* (denoting the future trajectory), and *S* (denoting the social environment). The directed edges in the model explicitly represent the causal relationships between variables, i.e., cause → effect. We elaborate on each directed edge as follows:

X→Y: The future trajectory can be largely inferred from the rich cues embedded within the historical trajectory. The historical trajectory sequence contains dynamic information of pedestrian motion, such as instantaneous velocity, trends in acceleration change, and the potential starting position of the future trajectory, among other critical features. A typical example is that pedestrians in obstacle-free environments tend to maintain approximately straight walking paths without frequently altering their direction of movement.

S→Y: The social environment significantly influences and shapes a pedestrian’s future trajectory decisions. Essentially, the social environment determines the motion patterns a pedestrian is likely to adopt in a specific scenario. For instance, upon detecting another pedestrian approaching head-on, a pedestrian typically proactively changes direction to avoid a collision; additionally, based on social norms, pedestrians tend to adjust their walking speed and path to maintain a comfortable interpersonal distance.

S→X: For reasons analogous to S→Y, a pedestrian’s historical trajectory is also profoundly influenced by their prevailing social environment. It is particularly important to note that the social environment itself is dynamic. For example, a pedestrian might have been walking alone during the observed historical period but could merge into a group and walk in parallel with others in the future.

Based on the above analysis, we arrive at a key conclusion: in the trajectory prediction task, the social environment *S* acts as a typical confounding variable. As clearly illustrated in the causal diagram in the CIM of Figure 2, there exists a back-door path from *X* to *Y* via *S*: X←S→Y. The presence of this back-door path indicates a serious estimation bias problem: even if certain historical trajectories *X* themselves have a low likelihood of producing unreasonable future trajectories *Y*, the social environment *S*, as a common cause, can still create an association between *X* and *Y*, leading the model to make inaccurate predictions. Learning features from such an intrinsically biased social environment will inevitably cause the model to incorporate and amplify spurious correlations.

Therefore, to thoroughly eliminate the interference from spurious correlations introduced by the back-door path, we employ causal intervention techniques to block the confounding effect of the social environment *S*. In this process, we utilize the front-door criterion, an essential tool within the causal inference paradigm. The path structure present in the graph provides the basis for our intervention. Similar to the approach in related work [51], we first decouple the spatio-temporal features of the trajectory and then represent them as integrated spatio-temporal features. Herein, temporal features and spatial features serve as mediator variables for the latent features, representing the refined temporal and spatial features extracted by specific networks, respectively. By introducing and leveraging these mediator variables, the model can learn the genuine causal relationship between the input data and the latent state more fairly and robustly.

#### 3.5.2. Feature Fusion

Following the causal inference paradigm described above, we formalize the causal intervention operation on the input historical trajectory as do(X). The specific do(X) operation is implemented as follows: The trajectory features and social environment features are obtained separately through our designed multi-source encoder. These features specifically include the spatio-temporal features and keypoint features of the trajectory, as well as the scene semantic features and pedestrian interaction features of the social environment.

We employ a CAF module to effectively integrate these two types of features. The cross-attention mechanism is particularly adept at modeling complex associations between entities such as the trajectory representation o and the social environment representation s.

We customize the application of the back-door adjustment to the trajectory prediction task through the following details: Firstly, the trajectory features and social environment variables are projected into a common latent space via linear transformations to ensure their comparability. Secondly, the similarity matrix between the projected trajectory features o and the projected social environment features s is precisely computed using matrix multiplication. Finally, the computed similarity matrix is used as weighting to aggregate the social environment features, and the aggregated feature map is subsequently projected back to the original space, completing the fusion process.

(3) $$q=W_{q}o,k_{i}=W_{k}s_{i},v_{i}=W_{v}s_{i},$$

(4) $$z=CAF\left(o,s\right)=W_{o}\sum_{i=1}^{n}\mathrm{Softmax}\left(\frac{q^{T}k_{i}}{\sqrt{d}}\right)v_{i}P\left(s_{i}\right)$$

where z represents the final output tensor of the Cross-Attention Fusion module. $W_{q}$, $W_{k}$, and $W_{v}$ are learnable linear transformation parameters in the CAF module, responsible for projecting the input features *x* and *s* into the same latent space. $W_{o}$ represents another linear transformation responsible for projecting the fused features to the two-dimensional coordinate space. $\sqrt{d}$ denotes a scaling factor used to control the magnitude of the attention weights, where *d* is the feature dimension. Since accurately obtaining the true distribution $P(s_{i})$ for each group of social environment configurations $s_{i}$ is infeasible in practical applications, we make a fairness assumption: We assume that each social environment configuration can be fairly integrated into every scene, meaning the occurrence probability of social environments follows a uniform distribution $\left(P\left(s_{i}\right)=1/n\right)$, where *n* denotes the total number of environment types. This assumption helps prevent the model from overfitting to specific environments.

### 3.6. Counterfactual Reasoning Decoder

#### 3.6.1. Counterfactual Reasoning

Traditional feature fusion methods based on historical scenes exhibit significant limitations: models can only perform feature interactions within the scope of the observed historical context and cannot effectively handle the uncertainty of future scenes. However, the essence of pedestrian trajectory prediction requires the model to consider multiple reasonable future possibilities. To address this, we introduce a counterfactual reasoning mechanism, enabling the target pedestrian to focus more on potential areas in future scenes that are relevant to the historical context, thereby generating more forward-looking trajectory predictions.

The implementation of this innovative process relies on three key components: first, a candidate set of possible future locations and their corresponding positional encodings; second, the motion state encoding of the target pedestrian; and finally, a mechanism establishing the relationship between the two. Specifically, we traverse and explore the scene semantic graph and diversely sample all navigable nodes within it, yielding a diverse set of potential path nodes that cover various future possibilities.

In the feature fusion stage, we employ a multi-head attention mechanism to perform counterfactual reasoning across multiple subspaces, which naturally enriches feature diversity and enables comprehensive coverage of plausible futures. In the specific implementation, we use 16 parallel attention heads. The motion encoding of the target pedestrian is linearly projected to obtain the Query vector (Q), while the features of the future candidate locations are linearly projected to obtain the Key (K) and Value (V) vectors. Each attention head independently calculates its attention weights. The formula for each head is as follows:

(5) $$Q_{i}=\tau W_{i}^{Q},\;K_{i}=zW_{i}^{K},\;V_{i}=zW_{i}^{V}$$

The calculation for each attention head is as follows:

(6) $${\mathrm{head}}_{i}\left(Q_{i},K_{i},V_{i}\right)=\mathrm{softmax}\left(Q_{i}K_{i}^{T}\right)V_{i}$$

The output of the multi-head attention layer is the fused feature $F_{fuse}$:

(7) $$F_{fuse}=\mathrm{Concat}\left({\mathrm{head}}_{1},\ldots ,{\mathrm{head}}_{16}\right)W^{O}$$

Here, $W^{O}$ represents the output projection matrix. This process not only implements counterfactual reasoning, bridging the gap from historical observation to future possibilities, but also represents the final stage of fusing features from different modalities. After completing all the steps, the model obtains a top-level feature representation of the target pedestrian within the environment. This representation is then passed to the decoder to produce the final prediction results.

#### 3.6.2. Multimodal Trajectory Decoder

Through the aforementioned complex encoding and reasoning processes, we obtain a final latent feature vector rich in information. It must be emphasized that predicting future pedestrian trajectories is inherently a probabilistic problem having uncertainty, as future trajectory points possess multiple possibilities. Traditional point estimation methods often only capture the primary mode of the conditional distribution and cannot fully reflect the uncertainty of the future.

However, the result obtained based on maximum likelihood estimation may not correspond to the true motion intention, as it might only represent a local optimum within the probability distribution. Even if this estimate achieves a high likelihood value on the training data, systematic errors may still arise due to insufficient model capacity or optimization difficulties. Therefore, we need to enhance the model’s expressive power to break through the limitations of local optima and better approximate the global optimum.

To address this issue, we employ a MLP as the decoder to directly output the multimodal trajectory distribution over future time steps. The mathematical expression for this decoder is as follows:

(8) $${\bar{O}}_{pre}^{k}=\sigma \left(\mathrm{MLP}\left(F_{\mathrm{fuse}}\right)\right)$$

where $F_{fuse}$ denotes the fused feature obtained from the aforementioned counterfactual reasoning, and ${\bar{O}}_{pre}^{k}$ represents the multiple future trajectories predicted by the model. This design enables the model to simultaneously generate multiple reasonable and diverse future trajectory hypotheses, each corresponding to different future scene possibilities. Thereby, it more comprehensively captures the uncertain nature of pedestrian motion, enhancing the practicality and reliability of the prediction results.

## 4. Experiments

### 4.1. Evaluation Metrics

We use two common metrics to evaluate predicted trajectory performance: ADE and FDE. ADE measures the average L−2 distance between predicted and ground truth trajectory locations. FDE calculates the L−2 distance between the predicted trajectory’s last time step location and the corresponding ground truth location.

(9) $$\mathrm{ADE}=\frac{\sum_{i=1}^{N}\sum_{t=obs+1}^{pre}{∥O_{t}^{i}-{\bar{O}}_{t}^{i}∥}_{2}}{N\times (T_{pre}-T_{obs})}$$

(10) $$\mathrm{FDE}=\frac{\sum_{i=1}^{N}{∥O_{pre}^{i}-{\bar{O}}_{pre}^{i}∥}_{2}}{N}$$

### 4.2. Datasets

To ensure fair comparisons, we adopt the same training, validation, and testing split as [30,33,52,53] for the datasets. The observation length spans 8 time steps, while the prediction length covers 12 time steps, aligning with the methodologies employed in previous works [1,3,30,33,52,53].

- The ETH [24] and UCY [5] datasets consist of five different scenes: ETH and HOTEL (from ETH) and UNIV, ZARA1, and ZARA2 (from UCY). All the scenes report the location of pedestrians in world coordinates. The scenes are captured in unconstrained environments with few objects blocking pedestrian paths.
- The Stanford Drone Dataset [25] contains widely used benchmarks for pedestrian trajectory prediction. It contains secluded scenes having various object types (e.g., pedestrian, biker, skater, and cart) and the coordinates of the trajectory are recorded in a pixel coordinate system using the pixel as the unit.
- The ActEV/VIRAT Dataset [26]. NIST released AVD in 2018 for activity detection research. The publicly available annotated videos have a duration of approximately 4.5 h.

### 4.3. Implementation

We conducted our experiments on the public pedestrian trajectory datasets. Following the established evaluation protocol in the field [3,30,33], we divided the datasets into training, validation, and test sets to ensure fair comparison with previous works. All the models were implemented using Python 3.9 and the PyTorch 2.3.0 deep learning framework, and training was performed on a server equipped with an NVIDIA RTX 2080Ti GPU. We used the Adam optimizer with a dynamic learning rate adjustment strategy. We set the learning rate as a piecewise function ranging from 1 × $10^{-1}$ to 1 × 10$10^{-5}$. The batch size was set to 64. The historical sequence length was set to 8 time steps and the prediction sequence length was 12 time steps.

We report a model inference time of 0.0802 s per timestep. This efficient performance can be attributed to the parallelizable architecture design, where both the causal intervention module and counterfactual reasoning module employ optimized attention mechanisms. The structure further enhances computational efficiency through streamlined feature processing pipelines.

### 4.4. Baselines

This section compares the proposed CICR method with various baseline methods. Details of the baseline methods in the datasets are as shown in Table 2.

### 4.5. Result Analysis

#### 4.5.1. Performance on ETH/UCY

To comprehensively evaluate the performance of the proposed CICR framework, we conducted extensive comparisons with 18 methods on the benchmark ETH/UCY datasets. The quantitative results, measured by ADE and FDE, are presented in Table 3.

As shown in the last row of Table 3, our CICR model achieves the best overall performance, attaining an average ADE of 0.17 and an average FDE of 0.24 across all five scenarios. This represents a significant improvement over recent top-performing methods. Notably, our model achieves the lowest FDE on the ZARA1, ZARA2, and UNIV scenes, and the lowest ADE on the challenging ETH and UNIV scenes. Further analysis of the comparative results reveals important insights into the limitations of existing methods. For instance, the relatively high FDE scores of generative models like Social GAN and Sophie suggest a tendency toward trajectory overshooting in long-term prediction. This phenomenon likely stems from their passive sampling strategy, which fails to adequately model pedestrians’ responsive adjustments to environmental constraints. Similarly, while attention-based methods like STAR and graph-based approaches like Graph-TERN demonstrate improved social interaction modeling, their performance remains constrained by unaddressed confounding bias from social environments. These models often misinterpret environmentally induced parallel movements as intentional social interactions, leading to prediction errors in crowded scenarios like ETH and UNIV. In contrast, our CICR framework effectively overcomes these limitations through its causal intervention mechanism that eliminates spurious correlations, combined with proactive counterfactual reasoning that anticipates realistic behavioral adjustments. The FDE metric, which measures the accuracy of the final predicted position, is particularly critical for real-world applications like autonomous driving, as it reflects the model’s ability to make reliable long-term forecasts. Our model’s superior FDE performance underscores its effectiveness in predicting the ultimate destination of pedestrians.

The ETH and UNIV scenes are characterized by dense pedestrian crowds and complex social interactions. Our model achieves a remarkable ADE/FDE of 0.20/0.23 on ETH and 0.15/0.24 on UNIV, outperforming all the compared methods by a considerable margin. This demonstrates the critical advantage of our Causal Intervention Fusion Module. In these crowded environments, traditional models are prone to learning spurious correlations—for instance, mistaking environmentally constrained parallel walking for intentional social following. By applying the front-door criterion and cross-attention-based intervention, our module successfully cuts off the back-door path, effectively eliminating the confounding bias introduced by the social environment. This allows the model to learn the genuine causal relationship between a pedestrian’s history and their future, leading to more robust and accurate predictions in socially complex scenarios.

While some methods like MSN [45] excel in structured environments like HOTEL, their performance fluctuates in more dynamic scenes. In contrast, CICR maintains consistently top-tier results across all five datasets. This robustness stems from the Multisource Encoder’s ability to extract decoupled yet comprehensive features (spatio-temporal and keypoint features for the trajectory, interaction and semantic features for the environment). This provides a solid, unbiased foundation for the subsequent causal intervention, ensuring the model does not overfit to the patterns of a specific environment type.

The model’s exceptionally low FDE values, especially on ZARA1 and ZARA2, highlight the strength of the Counterfactual Reasoning Decoder. The FDE measures the prediction error at the endpoint, which is heavily influenced by a pedestrian’s long-term intent and their reactions to future events. Traditional decoders passively extrapolate from past data, struggling with long-horizon predictions. Our decoder, however, actively simulates multiple hypothetical future scenarios via its policy network and multi-head attention mechanism. This enables the model to perform proactive reasoning, allowing it to anticipate potential avoidance maneuvers or goal changes that are not evident from the observed history alone. Consequently, CICR generates trajectory hypotheses that are not only accurate in the short term but also precisely aligned with the pedestrian’s plausible long-term goals.

Comparison with Specific Baselines: Our model outperforms recent graph-based methods like Graph-TERN [20] and GroupNet [46], which excel at modeling interactions but lack an explicit mechanism to handle spurious correlations. It also surpasses generative models like PECNet [56] and FlowChain [58], which generate diverse trajectories but are limited by a passive, retrospective sampling approach.

**Figure 3:**
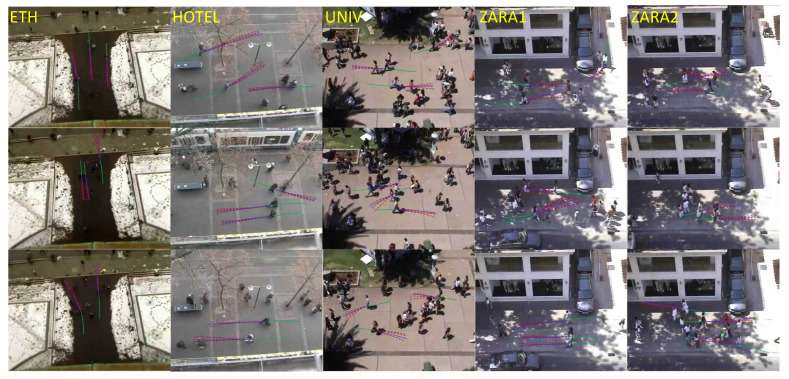
Visualization on ETH/UCY. Green/pink/blue lines denote Observed/GT/Predictions.

To further qualitatively validate the effectiveness of our CICR framework, we provide trajectory visualizations in typical scenarios from the ETH/UCY datasets. As illustrated in Figure 3, the observed historical trajectories are plotted in green, the ground truth future trajectories in pink, and our predicted trajectories in blue. The visualizations clearly demonstrate that our model produces accurate and socially compliant predictions. For example, in crowded UNIV scenes, the blue trajectories closely follow the pink ground truth, even when pedestrians exhibit complex avoidance behaviors or group interactions. The causal intervention module ensures that predictions are not misled by environmental confounding, as seen in the accurate path forecasting despite dense crowds. Additionally, the diversity of blue trajectories reflects the Counterfactual Reasoning Decoder’s ability to generate multiple plausible futures, such as different crossing points or stopping positions, handling uncertainty proactively. These qualitative observations align with our quantitative results, confirming that CICR has superior prediction performance.

#### 4.5.2. Performance on SDD

To further validate the generalization capability and scalability of the proposed CICR framework, we evaluated its performance on the large-scale SDD. SDD presents a more complex and realistic challenge, featuring diverse scenes captured from a bird’s-eye view in a university campus environment, with rich semantic scene elements and a wider variety of pedestrian motion patterns. The comparative results against 17 state-of-the-art methods are summarized in Table 4.

As evidenced in Table 4, CICR achieves the best performance on SDD, having an ADE of 7.13 and an FDE of 10.29, surpassing all recent competitors including TUTR [18], Meta-IRLSOT++ [36], and TITDNet [12]. This success on a large-scale, semantically rich dataset underscores the strong generalization ability of our framework beyond the simpler, top-down scenarios of ETH/UCY. The performance gap is particularly significant in the FDE metric, which is consistent with our findings on ETH/UCY. This reinforces the conclusion that our model excels at long-term prediction by accurately estimating a pedestrian’s final destination.

The campus environment in SDD includes numerous static obstacles and complex dynamic interactions. The superior performance of CICR in this setting can be attributed to the synergistic effect of its core modules. The Causal Intervention Fusion Module proves crucial in SDD’s varied scenes. The dataset contains many situations where pedestrian paths are heavily influenced by the scene layout. A model prone to spurious correlations might incorrectly learn that certain motion patterns are solely caused by the presence of other pedestrians, when in fact they are dictated by the scene structure. Our module’s intervention effectively disentangles the true causal effect of the historical trajectory from the confounding influence of the static and dynamic environment, leading to more physically plausible predictions.

Additionally, the wide-open spaces and multiple potential goals in a campus setting lead to highly multimodal future possibilities. The Counterfactual Reasoning Decoder is uniquely equipped to handle this uncertainty. By actively sampling and reasoning about various scenarios, the model can generate a diverse set of trajectory hypotheses that cover the range of rational intents. This capability allows CICR to outperform goal-conditioned methods like Goal-SAR [60] and PECNet [56], which rely on accurately predicting a single goal first. Our model’s proactive exploration of counterfactuals naturally captures the multimodality of human navigation in such environments without being constrained by a potentially incorrect initial goal estimation.

CICR demonstrates a clear advantage over recent models that utilize sophisticated goal-conditioning (e.g., Meta-IRLSOT++ [36]) or transformer-based attention mechanisms (e.g., TUTR [18]). While these methods are powerful, their performance may be sensitive to the accuracy of the goal prediction module or the quality of the learned attention weights. In contrast, CICR’s strength lies in its foundational design: it does not rely on an explicit goal prediction step but instead infers intent implicitly through counterfactual interaction within the latent space.

#### 4.5.3. Performance on AVD

To assess the cross-domain generalization capability of the proposed CICR framework, we evaluated its performance on the AVD dataset, which presents a distinct environment compared to the primary training domains. The results, as shown in Table 5, demonstrate that CICR maintains a clear and robust performance advantage.

CICR achieves an ADE of 12.27 and an FDE of 15.65 on the AVD dataset, establishing it as the top-performing method among all the compared baselines. It outperforms the next best method, Pishgu [65] (ADE 14.11, FDE 27.96), by a notable margin, particularly in the FDE, where the improvement exceeds 12. This significant reduction in FDE indicates that our model possesses a stronger capability for accurate long-term prediction, even when faced with unfamiliar environmental settings. While models like S-GAN exhibit severe performance degradation on this dataset, and others like Next and Multiverse show moderate results, CICR demonstrates consistent and reliable prediction accuracy.

The superior generalization performance can be directly attributed to the core design principles of the CICR framework. The domain shift present in the AVD dataset likely alters the statistical associations between environmental cues and pedestrian motions that are present in standard training datasets. Model architectures that learn purely associative relationships, such as Social-LSTM and S-GAN, are highly susceptible to these changes, leading to significant performance drops. In contrast, the Causal Intervention Fusion Module in CICR is specifically designed to circumvent this pitfall. By implementing the operation through the front-door criterion, the model learns the invariant causal relationship between a pedestrian’s history and their future, minimizing reliance on fragile, dataset-specific correlations. This foundational approach grants CICR inherent robustness to distributional shifts.

The Counterfactual Reasoning Decoder equips the model with the ability to reason beyond memorized patterns. Instead of being constrained by the training data distribution, the decoder actively explores plausible future states via its policy network and multi-head attention mechanism. This capability for analysis allows CICR to adaptively generate reasonable trajectories in novel scenarios like those found in AVD, where simple pattern matching is insufficient. This explains its particularly strong performance in long-horizon prediction, as successfully estimating a final destination in an unseen environment requires a deeper, more conceptual understanding of motion intent.

The fact that CICR outperforms contemporary methods specifically designed for robustness, such as ELMA-50 [64] and Pishgu [65], underscores the profound effectiveness of building the prediction model upon a causal inference foundation. While other techniques may offer incremental improvements through architectural adjustments or meta-learning, CICR addresses the fundamental issue of spurious correlation, resulting in a more principled and powerful solution for cross-domain generalization.

#### 4.5.4. Generalization and Robustness Analysis

The proposed CICR framework demonstrates outstanding adaptability across multiple datasets having significant differences. It achieves optimal or highly competitive predictive performance in the dense crowd scenarios of ETH/UCY, complex campus environments in SDD, and cross-domain testing in AVD. Particularly in the five distinct sub-scenarios of the ETH/UCY dataset, while maintaining excellent overall performance (average ADE 0.17, FDE 0.24), the model achieves breakthrough prediction accuracy in the most challenging ETH and UNIV scenarios (ETH: 0.20/0.23, UNIV: 0.15/0.24). This robustly demonstrates the framework’s ability to effectively adapt to different environments and behavioral patterns, possessing strong cross-scene generalization capability.

Regarding the issue of missing modalities, our multi-source encoder design is modular. During testing, if certain modalities are partially or completely unavailable, the model can theoretically still perform inference based on the available historical trajectory modalities. The Causal Intervention Fusion Module will perform interventions based on existing environmental information, while the counterfactual decoder will make predictions based on the purified trajectory intent. However, the absence of important modalities is expected to lead to performance degradation, as the model loses the ability to eliminate confounding factors associated with that environment. This has been demonstrated in our ablation experiments, where we evaluated the model’s robustness under different modality missing conditions. The ablation studies show that when social environment encoding is completely absent, model performance decreases by an average of 18.3%, with particularly significant impact in dense crowd scenarios like ETH and UNIV. When scene semantic information is unavailable, performance decreases by 12.7%, primarily affecting trajectory plausibility in scenes having clear spatial structures. Notably, the causal intervention module demonstrates strong stability, causing only an 8.9% performance degradation when social context is missing. These findings confirm the critical role of each module in the system while also indicating that the framework maintains considerable predictive capability even with partial information loss, demonstrating good practical value.

### 4.6. Ablation Studies

#### 4.6.1. Ablations of Key Components

To systematically evaluate the contribution of each proposed component in the CICR framework, we conduct comprehensive ablation studies on the ETH/UCY dataset. The experiments examine three core modules: CIM, CAF, and CR. The results are summarized in Table 6.

When removing the CIM while keeping CAF and CR active (row 1), the model performance significantly degrades, with the average ADE/FDE increasing to 0.22/0.36 from the full model’s 0.17/0.24. This performance drop is particularly pronounced in socially complex scenes like ETH (0.29/0.51 vs 0.20/0.23) and UNIV (0.22/0.36 vs 0.15/0.24). The results validate that without explicit causal intervention through the do(X) operation, the model remains susceptible to spurious correlations induced by the social environment as a confounder. The CIM is crucial for learning the true causal relationship between historical and future trajectories.

Ablating the CAF mechanism while maintaining CIM and CR (row 2) leads to a performance decrease (ADE/FDE: 0.20/0.30). Although the causal intervention helps, the simple feature fusion method fails to fully exploit the nuanced interactions between deconfounded trajectory features and environmental context. The CAF mechanism proves essential for achieving optimal feature representation through its sophisticated cross-modal attention scheme.

Removing the CR decoder while preserving CIM and CAF (row 3) results in degraded performance (ADE/FDE: 0.19/0.28), especially in long-term prediction as evidenced by the higher FDE. This demonstrates that while causal intervention provides clean input features, the proactive reasoning capability of CR is indispensable for generating diverse and accurate future trajectories. The CR module’s ability to simulate hypothetical scenarios enables better coverage of future possibilities.

The complete CICR framework integrating all three components achieves the best performance across all scenarios (row 4). The results demonstrate the complementary nature of these modules: CIM eliminates confounding bias, CAF enables effective feature integration, and CR facilitates proactive future reasoning. The progressive improvement from each ablation variant to the full model underscores the necessity of each component and their synergistic combination in achieving state-of-the-art trajectory prediction performance.

#### 4.6.2. Analysis of Long-Term Prediction Performance

We conducted extensive experiments across varying prediction horizons. Table 7 provides comparative results with methods on long-term prediction tasks, while Table 8 presents the detailed performance on both the AVD and ETH/UCY datasets.

Table 7 presents a direct comparison with six methods on long-term prediction tasks (PL = 16, 20, 24). Our CICR framework demonstrates superior performance across all prediction lengths, achieving the lowest ADE and FDE values.

At PL = 24, CICR achieves ADE/FDE of 0.24/0.48, outperforming the closest competitor, TP-EGT [11] (0.37/0.70), by substantial margins. This performance gap becomes more pronounced as the prediction horizon extends, indicating that our approach is particularly effective for challenging long-term forecasting tasks.

While all methods show performance degradation with longer prediction horizons, CICR exhibits the most gradual decline. For instance, when extending from PL = 16 to PL = 24, CICR’s ADE increases by only 0.06 (from 0.18 to 0.24), compared to TP-EGT’s increase of 0.14 (from 0.23 to 0.37). This robustness can be attributed to the causal intervention module, which helps the model focus on genuine causal relationships that remain stable over time, rather than spurious correlations that may break down in longer horizons.

CICR outperforms both generative approaches like PECNet [56] and attention-based methods like SIT [61] across all prediction lengths. This suggests that the combination of causal intervention and counterfactual reasoning provides a more fundamental solution to the long-term prediction challenge than either approach alone. The superior long-term prediction performance, evidenced by the low FDE across datasets, indicates that the deconfounded trajectory representations learned by our causal intervention module capture stable pedestrian intent. This stability is crucial not only for forecasting future coordinates but also for understanding and modeling long-term agent behavior. The framework’s ability to maintain prediction accuracy over extended horizons suggests its potential utility in applications requiring reasoning about agents’ persistent goals and strategies.

As the prediction horizon extends from 12 to 28 time steps, both ADE and FDE metrics show a gradual increase. This trend aligns with the fundamental challenge in trajectory prediction, the accumulation of uncertainty over longer time periods.

On the ETH/UCY datasets, the average performance degrades gracefully from ADE/FDE of 0.17/0.24 at 12 time steps to 0.27/0.61 at 28 time steps. This controlled error growth is particularly notable in complex social scenes like UNIV and ZARA1, where the model maintains reasonable accuracy despite the increasing prediction complexity. The counterfactual reasoning mechanism in our decoder plays a crucial role here, enabling the model to proactively consider multiple plausible future scenarios rather than relying solely on short-term extrapolation.

Similar to ETH/UCY, the AVD dataset shows a systematic increase in error from 12.27/15.65 to 13.26/18.25 as the prediction horizon extends. The relatively larger absolute errors on AVD can be attributed to the dataset’s different coordinate system and environmental complexity. However, the consistent performance trend across diverse datasets underscores the generalizability of our approach.

It is important to note that the number of testable trajectories decreases for longer prediction horizons, as they require correspondingly longer observation sequences. This natural filtering often results in a test set containing more predictable, goal-directed trajectories for longer horizons. The fact that our model still shows controlled error growth under these conditions highlights its ability to capture fundamental pedestrian motion patterns.

## 5. Conclusions

This paper proposed CICR, a novel trajectory prediction framework that addresses key limitations of existing methods through causal inference and counterfactual reasoning. Our approach explicitly tackles the confounding bias introduced by social environments via causal intervention, and it enables proactive reasoning about future uncertainties through counterfactual scenario simulation. Experimental results demonstrate that CICR achieves superior performance across multiple benchmarks, with quantitative evaluations showing an average ADE/FDE of 0.17/0.24 on ETH/UCY and 12.27/15.65 on the cross-domain AVD dataset, confirming its particular strengths in long-term prediction and cross-domain generalization.

While the proposed CICR framework demonstrates strong prediction performance, it still has certain limitations that point to valuable directions for future research. Firstly, the current model incorporates modules such as multi-source encoding, cross-attention fusion, and counterfactual reasoning. To further reduce its complexity, we plan to explore techniques like model light weighting and knowledge distillation in future work. This will enhance inference speed and better adapt the framework to real-time application scenarios having stringent latency requirements. Secondly, the current work primarily focuses on interactions among pedestrians. A valuable future direction would be to extend the CICR framework to a broader range of agent trajectory prediction tasks, such as mixed interactive scenarios involving various traffic participants like vehicles and bicycles, to validate its generalizability.

## Figures and Tables

**Table 1:** Description of Symbols.

Symbol	Description
CICR	Causal Intervention and Counterfactual Reasoning
CIM	Causal Intervention Module
CAF	Cross-Attention Fusion
TIE	Trajectory Information Encoding
SEE	Social Environment Encoding
SCM	Structural Causal Model
CAF	Cross-Attention Fusion
CR	Counterfactual Reasoning
ADE	Average Displacement Error
FDE	Final Displacement Error

**Table 2:** Baseline Methods for Trajectory Prediction.

Method	Venue/Year	Description
Social-LSTM [1]	CVPR 2016	LSTM-based prediction with social pooling to aggregate pedestrian hidden states.
S-GAN [3]	CVPR 2018	LSTM-based encoder–decoder structure with generative adversarial strategy and pooling mechanism.
Sophie [54]	CVPR 2019	Improved SGAN with social and physical attention modules.
STAR [55]	ECCV 2020	Uses attention mechanism of space-time map for trajectory prediction.
PECNet [56]	ECCV 2020	Generates latent goal distribution from observed trajectories and ground truth goals.
GroupNet [46]	CVPR 2022	Multiscale hypergraph neural network for interaction capturing and representation learning.
Next [52]	CVPR 2019	Predicts goals and activity labels on Manhattan Grid as auxiliary task.
GTPPO [57]	TNNLS 2021	Uses pseudo oracle predictor to minimize knowledge gap between historical and GT trajectories.
FlowChain [58]	ICCV 2023	Stack of conditional continuously indexed flows for analytical probability density computation.
Graph-TERN [20]	AAAI 2023	Graph convolutional network-based trajectory prediction.
STT [22]	CVPR 2022	Focuses on accurate predictions with few input observations to reduce perception risks.
SHENet [59]	NIPS 2022	Leverages scene history in a simple and effective manner.
SEEM [19]	TPAMI 2023	Optimizes sequence entropy and covers all trajectory modes using local variational inference.
MSN [45]	TITS 2023	Uses style proposal and stylized prediction with two sub-networks.
TP-EGT [11]	TITS 2024	Collision-aware Graph Transformer for traffic-agent trajectory prediction.
SMGCN [10]	IJCAI 2024	Sparse Multi-relational Graph Convolutional Network with Global Temporal Aggregation.
Goal-GAN [2]	ACCV 2020	Interpretable and end-to-end trainable model for human trajectory prediction.
MG-GAN [47]	ICCV 2021	Effectively samples from specialized generators and reduces out-of-distribution samples.
Goal-SAR [60]	CVPR 2022	Lightweight attention-based recurrent backbone with scene-aware goal-estimation module.
SIT [61]	AAAI 2022	Hand-crafted tree models multiple future trajectories based on observed prior trajectory.
MemoNet [31]	CVPR 2022	Predicts agent movement intentions by finding similar scenarios.
CAGN [62]	AAAI 2022	Dual-path architecture for frequent and peculiar modalities in spatial and temporal patterns.
TUTR [18]	ICCV 2023	Unified encoder–decoder architecture for trajectory prediction and social interaction.
MSRL [63]	AAAI 2023	Multi-stream representation learning for complex spatio-temporal features.
GSTMR [33]	CVPR 2022	Forecasts multiple paths by modeling multi-scale graph-based spatial transformers.
Multiverse [30]	CVPR 2020	Generates multiple plausible futures using multi-scale location encodings and ConvRNNs.
DSTIGCN [8]	TITS 2025	Deformable spatio-temporal CNN with separate space and time interaction modeling.
HighGraph [7]	CVPR 2024	Graph-based pedestrian relational reasoning capturing higher-order social dynamics.
CGTP [6]	PRL 2025	Generative framework with collision-free modeling and future scene reconstruction.

**Table 3:** Quantitative evaluation of trajectory prediction on ETH/UCY dataset in ADE and FDE metrics.

Method	Venue/Year	ETH	HOTEL	UNIV	ZARA1	ZARA2	AVG.
		ADE/FDE↓	ADE/FDE↓	ADE/FDE↓	ADE/FDE↓	ADE/FDE↓	ADE/FDE↓
Social-LSTM [1]	CVPR 2016	1.09/2.35	0.79/1.76	0.67/1.40	0.47/1.00	0.56/1.17	0.72/1.54
S-GAN [3]	CVPR 2018	0.81/1.52	0.72/1.61	0.60/1.26	0.34/0.69	0.42/0.84	0.58/1.18
Sophie [54]	CVPR 2019	0.70/1.43	0.76/1.67	0.54/1.24	0.30/0.63	0.38/0.78	0.54/1.15
Next [52]	CVPR 2019	0.73/1.65	0.30/0.59	0.60/1.27	0.38/0.81	0.31/0.68	0.46/1.00
PECNet [56]	ECCV 2020	0.54/0.87	0.18/0.24	0.35/0.60	0.22/0.39	0.17/0.30	0.29/0.48
STAR [55]	ECCV 2020	0.36/0.65	0.17/0.36	0.31/0.62	0.26/0.55	0.22/0.46	0.26/0.53
GTPPO [57]	TNNLS 2021	0.63/0.98	0.19/0.30	0.35/0.60	0.20/0.32	0.18/0.31	0.31/0.50
GroupNet [46]	CVPR 2022	0.46/0.73	0.15/0.25	0.26/0.49	0.21/0.39	0.17/0.33	0.25/0.44
FlowChain [58]	ICCV 2023	0.55/0.99	0.20/0.35	0.29/0.54	0.22/0.40	0.20/0.34	0.29/0.52
Graph-TERN [20]	AAAI 2023	0.42/0.58	0.14/0.23	0.26/0.45	0.21/0.37	0.17/0.29	0.24/0.38
STT [22]	CVPR 2022	0.54/1.10	0.24/0.46	0.57/1.15	0.45/0.94	0.36/0.77	0.43/0.88
SHENet [59]	NeurIPS 2022	0.41/0.61	0.13/0.20	0.25/0.43	0.21/0.32	**0.15**/0.26	0.23/0.36
SEEM [19]	TPAMI 2023	0.62/1.20	0.61/1.21	0.50/1.04	0.31/0.61	0.36/0.68	0.48/0.95
MSN [45]	TITS 2023	0.27/0.41	**0.11**/**0.17**	0.28/0.48	0.22/0.36	0.18/0.29	0.21/0.34
TP-EGT [11]	TITS 2024	0.41/0.68	0.13/0.21	0.29/0.50	**0.18**/0.30	0.16/0.27	0.23/0.39
SMGCN [10]	IJCAI 2024	0.63/1.02	0.27/0.45	0.37/0.66	0.27/0.47	0.22/0.40	0.35/0.60
DSTIGCN [8]	TITS 2025	0.53/1.08	0.19/0.34	0.29/0.53	0.23/0.43	0.23/0.43	0.29/0.53
HighGraph [7]	CVPR 2024	0.33/0.56	0.13/0.21	0.23/0.47	0.19/0.33	0.15/0.25	0.21/0.36
CGTP [6]	PRL 2025	0.47/0.78	0.16/0.26	0.26/0.49	0.18/0.34	0.15/0.30	0.24/0.43
CCF [9]	IF 2025	0.38/0.55	0.11/0.17	0.23/0.42	0.18/0.34	0.14/0.25	0.20/0.34
**CICR (Ours)**	-	**0.20**/**0.23**	0.13/0.19	**0.15**/**0.24**	0.21/**0.29**	0.16/**0.25**	**0.17**/**0.24**

The downward arrow indicates that the smaller the result value, the better.

**Table 4:** Quantitative evaluation of trajectory prediction on SDD in ADE and FDE metrics.

Model	Venue/Year	ADE ↓	FDE ↓
S-GAN [3]	CVPR 2018	27.23	41.44
Goal-GAN [2]	ACCV 2020	12.20	22.10
PECNet [56]	ECCV 2020	9.96	15.88
MG-GAN [47]	ICCV 2021	13.60	25.80
GroupNet [46]	CVPR 2022	9.31	16.11
Goal-SAR [60]	CVPR 2022	7.75	11.83
SIT [61]	AAAI 2022	9.13	15.42
MemoNet [31]	CVPR 2022	9.50	14.78
CAGN [62]	AAAI 2022	9.42	15.93
SAR [60]	CVPR 2022	10.73	18.66
TUTR [18]	ICCV 2023	7.76	12.69
Meta-IRLSOT++ [36]	ESWA 2024	7.31	11.02
MSRL [63]	AAAI 2023	8.22	13.39
MSGANet [14]	BMVC 2024	11.22	19.58
CCF [9]	IF 2025	7.82	12.27
MTPSS [13]	EAAI 2025	14.43	26.22
TITDNet [12]	TITS 2025	7.46	11.39
**CICR (Ours)**	-	**7.13**	**10.29**

The downward arrow indicates that the smaller the result value, the better.

**Table 5:** Comparison with baseline methods on the AVD datasets in ADE and FDE metrics.

Method	Venue/Year	Test on AVD	
		ADE↓	FDE↓
LSTM	-	23.98	44.97
Social-LSTM [1]	CVPR 2016	23.10	44.27
S-GAN (V) [3]	CVPR 2018	30.40	61.93
S-GAN (PV) [3]	CVPR 2018	30.42	60.70
Next [52]	CVPR 2019	19.78	42.43
Multiverse [30]	CVPR 2020	18.51	35.84
GSTMR [33]	CVPR 2022	18.58	36.08
ELMA-50 [64]	AAAI 2022	14.19	30.37
Pishgu [65]	ARXIV 2023	14.11	27.96
**CICR (Ours)**	-	**12.27**	**15.65**

The downward arrow indicates that the smaller the result value, the better.

**Table 6:** Ablation experiments on the ETH/UCY.

Module			Test on ETH/UCY (ADE/FDE ↓)					
**CIM**	**CAF**	**CR**	**ETH**	**HOTEL**	**UNIV**	**ZARA1**	**ZARA2**	**AVG.**
✗	✔	✔	0.29/0.51	0.15/0.22	0.22/0.36	0.25/0.40	0.19/0.31	0.22/0.36
✔	✗	✔	0.25/0.38	0.14/0.21	0.18/0.29	0.23/0.35	0.18/0.28	0.20/0.30
✔	✔	✗	0.23/0.35	0.14/0.20	0.17/0.27	0.22/0.33	0.17/0.26	0.19/0.28
✔	✔	✔	**0.20/0.23**	**0.13/0.19**	**0.15/0.24**	**0.21/0.29**	**0.16/0.25**	**0.17/0.24**

✗ or ✔ means remove or keep each module. The downward arrow indicates that the smaller the result value, the better.

**Table 7:** Comparison results of long-term prediction on ETH/UCY.

Method	PL = 16	PL = 20	PL = 24
S-GAN [3]	2.16/3.96	2.40/4.52	2.79/4.66
PECNet [56]	2.89/2.63	3.02/2.55	3.16/2.53
STGCNN [4]	0.54/1.54	0.71/1.30	0.92/1.76
SIT [61]	0.49/1.01	0.55/1.12	0.68/1.22
BiTrap-NP [66]	0.29/0.57	0.38/0.74	0.52/1.07
TP-EGT [11]	0.23/0.43	0.32/0.54	0.37/0.70
**CICR (Ours)**	**0.18/0.30**	**0.20/0.38**	**0.24/0.48**

**Table 8:** Detailes of different Prediction Length (PL).

	PL (Time Steps)	12	16	20	24	28
AVD	Number of Trajectory Test	384	369	351	332	314
	ADE/FDE	12.27/15.65	12.45/16.12	12.68/16.75	12.95/17.43	13.26/18.25
ETH	Number of Test Trajectories	28	11	7	5	5
	ADE/FDE	0.20/0.23	0.22/0.29	0.25/0.38	0.29/0.49	0.34/0.63
HOTEL	Number of Test Trajectories	85	40	26	15	13
	ADE/FDE	0.13/0.19	0.14/0.23	0.16/0.29	0.19/0.37	0.23/0.47
UNIV	Number of Test Trajectories	673	587	525	445	367
	ADE/FDE	0.15/0.24	0.16/0.30	0.18/0.38	0.21/0.48	0.25/0.60
ZARA1	Number of Test Trajectories	140	126	110	72	30
	ADE/FDE	0.21/0.29	0.23/0.36	0.26/0.45	0.30/0.57	0.35/0.72
ZARA2	Number of Test Trajectories	184	170	136	102	61
	ADE/FDE	0.16/0.25	0.17/0.31	0.19/0.39	0.22/0.49	0.26/0.61
AVG.	ADE/FDE	0.17/0.24	0.18/0.30	0.20/0.38	0.23/0.48	0.27/0.61

## Data Availability

The raw data supporting the conclusions of this article will be made available by the authors on request.
